# Editorial: Adopting drug repurposing to overcome drug resistance in cancer

**DOI:** 10.3389/fcell.2023.1191682

**Published:** 2023-04-28

**Authors:** Eswar Shankar, Vish Subramaniam, Dharmaraja Allimuthu

**Affiliations:** ^1^ Department of Internal Medicine, Division of Medical Oncology, The Ohio State University Comprehensive Cancer Center, Columbus, OH, United States; ^2^ Department of Mechanical and Aerospace Engineering, The Ohio State University, Columbus, OH, United States; ^3^ Department of Chemistry, Indian Institute of Technology Kanpur, Kanpur, Uttar Pradesh, India

**Keywords:** cancer drug resistance, drug repurposing, FDA-approved drugs, natural product, combination therapy

## Introduction

**FIGURE 1:**
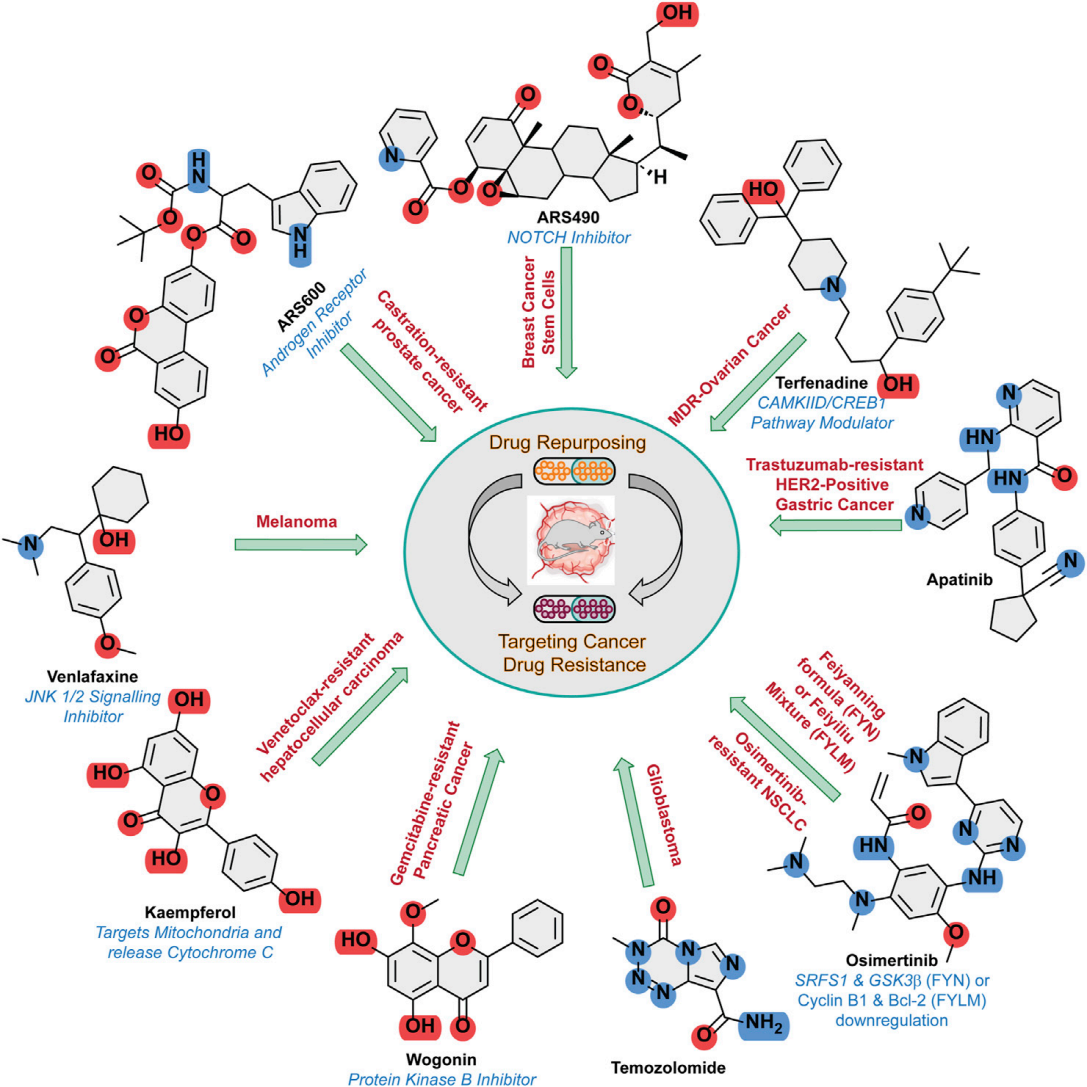
Summary of repurposed small molecules and their mechanism of actions to tackle drug resistance in the panel of cancers mentioned.

Despite significant technological advances the etiology of cancer and mechanism disease progression, and their translation into therapeutic benefits has been considerably slow. Traditional drug discovery efforts employing unbiased or target-based approaches involving natural products or small-molecule screening have created several therapeutics, but the entire process is tedious. Drug repurposing, also called drug repositioning, reprofiling, or retasking, identifies opportunities to use approved or investigational drugs that are outside the scope of the original medical indications (Ashburn and Thor, 2004). This strategy can be advantageous over developing an entirely new drug or formulation for a condition. It lowers the risk of failure as the repurposed drug’s safety has already been determined and found to be safe in preclinical models and humans through completed early-stage trials; thus, from a safety point of view, the drug is less likely to fail in subsequent efficacy trials (Breckenridge and Jacob, 2019). Drug resistance is a recurrent issue in oncology (Maxmen, 2016; Dharmaraja, 2017; Nikolaou et al., 2018) and researchers are actively pursuing innovative strategies to mitigate its impact. These approaches encompass a range of interventions, including immuno-oncological treatments that elicit the immune system’s response to target cancer cells (Dawe et al., 2020), combination therapies employing multiple drugs to attack cancer cells at different levels (Obenauf, 2022), and precision medicine that focuses on the molecular pathways underlying drug resistance to optimize treatment outcomes (Tsimberidou et al., 2020). These novel techniques aim to surmount the challenges of drug resistance in cancers and enhance patient outcomes. Repurposing drugs for cancer treatment has emerged as an increasingly attractive strategy as it can reduce the time to regulatory approval (Bertolini et al., 2015; Clohessy and Pandolfi, 2015; Corsello et al., 2017; Pantziarka, 2017; Pushpakom et al., 2019). In this Research Topic, we have collated research reports exploring the utility of organic small molecules, natural products, Chinese herbal medicines, and antibodies as combinatorial therapies to target drug resistance in cancers (Figure 1).

### Small molecules in combination therapy for cancer treatment

Small molecules with promising anticancer effects have a high possibility of becoming commercialized. Therefore, there is growing enthusiasm for exploring small molecules to advance the potency of existing anticancer therapeutics. A report by Chandrasekaran et al. has identified a tyrosinated-urolithin A derivative, ARS-600, as an effective inhibitor (EC_50_: <920 nM) of castration-resistant prostate cancer (CRPC) in xenografted castrated and non-castrated mice. The mechanism of the lead molecules was found to be the ubiquitination of the androgen receptor and its splice variant ARV7 resulting in signal inhibition in CRPC cells. Similarly, Saran et al. have identified a Withaferin A analog, ASR490, as an inhibitor of NOTCH-NICD (an active form of Notch1) in breast cancer stem cells at nanomolar concentrations. ASR490 triggers autophagy in cells to prevent cancer progression in *in vivo* xenograft models. High-throughput screening of bioactive molecules could help with the rapid identification of potential therapeutics and could provide insights into the mechanism of action. Huang et al. performed a quantitative high-throughput combinational screening of the LOPAC library (1,280 molecules, Sigma) and identified terfenadine (TFD) as a potential sensitizer of multidrug-resistant (MDR) ovarian cancer cells to doxorubicin. Here TFD molecule exerts a synergistic effect with doxorubicin against the survival of MDR ovarian cancer. They established that the mechanism of TFD function is not the manipulation of its canonical targets (Histamine receptor 1 or ether-a-go-go-related gene (hERG) channel), but direct modulation of the calcium-mediated signaling (CAMKIID/CREB1) pathway. Another small molecule-based combination therapeutic strategy with a flavonoid natural product, wogonin, was studied by Zhang et al. They show that gemcitabine-resistant pancreatic cancer cells became sensitive to gemcitabine when co-treated with wogonin. Subsequent mechanism-of-action analysis revealed suppression of the protein kinase B (Akt) signaling pathway with wogonin treatment resulting in apoptosis induction *in vivo*. Niu et al. have demonstrated that an antidepressant and antineoplastic drug, venlafaxine, could be used to control melanoma in mouse models. Venlafaxine induced apoptosis in melanoma through its interaction with JNK1/2 signaling to promote translocation of Nur77 to mitochondria, which triggered the activation of Bcl-2, cleaved caspase-3, and poly-ADP ribose polymerase (PARP) in mouse models.

### Natural products and herbal medicines that help overcome drug resistance

Natural products are a rich source of structurally diverse chemical scaffolds for the screening and identification of unique therapeutic candidates. This should not be a surprise given the successful development of taxanes as chemotherapeutic agents since the 1960s (Weaver, 2014). Chen et al. explored the therapeutic potential of kaempferol (KPL), a flavonoid natural product isolated from persimmon leaves. Here, they evaluate the efficacy of KPL on resensitizing drug-resistant hepatocellular carcinoma (HepG2) cells to venetoclax (ABT-199), a therapeutic approved to treat leukemia. The combination of KPL with ABT-199 demonstrated the induction of apoptosis through the disruption of mitochondrial membrane potential and the release of cytochrome C into mitochondria and cytoplasm, triggering apoptosis. The downstream apoptotic signature was observed in the reduction in anti-apoptotic proteins such as Bcl-2, Bcl-xL, and Mcl-1 and the upregulation of cleaved caspase. Traditional Chinese medicines have been used for the treatment of cancer for several decades. For example*,* Sang et al. showed that feiyanning formula (FYN), a Chinese herbal medicine cocktail prescribed for the treatment of lung cancer, could be exploited for targeting osimertinib-resistant non-small cell lung cancer (NSCLC). FYN has been shown to downregulate SRSF1 and GSK3β and thereby modulate the Wnt/β-catenin pathway in osimertinib-resistant NSCLC, HCC827OR, and PC9OR cells. Further, FYN (250 μg/mL) was found to elicit a synergistic effect with osimertinib (4 µM), an epidermal growth factor receptor (EGFR) tyrosine kinase inhibitor, on osimertinib-resistant NSCLC cells after 48 h of treatment and to prevent cell proliferation and migration; the combination suppressed tumor growth in mouse xenograft models of lung adenocarcinoma. Another Chinese herbal formula, feiyiliu mixture (FYLM), was described as sensitizing mutant EGFR-NSCLC to osimertinib by Shi et al. The major components of FYLM, characterized by mass spectrometry, included several antioxidant components such as quercetin, apigenin, formononetin, scutellarin, and oleanolic acid. EGFR-Del19/T790M/C797S mutant NSCLC is resistant to osimertinib; when combined with FYLM, the cells underwent apoptosis. It was shown that FYLM reduced EGFR phosphorylation and downregulated cyclin B1 and Bcl-2 while upregulating levels of cleaved caspase-3 to promote apoptosis *in vivo*. Peng et al. performed a metadata analysis of 31,263 patients treated with 16 Chinese herbal injections (CHIs) in combination with Western medicines. In a detailed analysis of 16 CHIs used either alone or in combination to treat cancer in China, a few were shown to be exceptionally beneficial in terms of reducing gastrointestinal adverse reactions, the incidence of thrombocytopenia, and the incidence of leukopenia when combined with Western medicines.

### Antibody and nanocarrier systems targeting drug resistance

Antibodies and antibody-drug conjugates (ADCs) that have been approved by the FDA for use in cancer treatment are promising classes of cancer therapies and precision medicines. However, the inherent reduction in activity due to *de novo* resistance development in the phenotype has prompted the combination therapies evolution of ADCs with chemotherapeutics. Lv et al. describe a case report of overcoming trastuzumab resistance in human epidermal growth factor receptor 2 (HER2)-positive gastric cancer by treating it with a triple regimen of apatinib and camrelizumab with trastuzumab. This issue also includes a research article on nanoparticle-based drug delivery systems for cancer treatment. Here, Wu et al. have developed a trifunctional, covalent nanocarrier system (Pep-1@PDA-TMZ) as a chemotherapeutic and photothermal therapeutic (PTT) to treat glioblastoma. This Pep-1@PDA-TMZ is based on dopamine polymeric nanoparticles covalently linked to a glioblastoma drug, temozolomide (TMZ), and Pep-1, a cell-penetrating peptide for enabling blood-brain barrier (BBB) breach. The conjugate Pep-1@PDA-TMZ was shown to penetrate cells effectively and was delivered specifically to the therapeutic site with a 77% inhibition of U87 cells in tumor-bearing nude mice *in vivo* recorded upon irradiation. This Research Topic also includes reviews by Xia et al. and Wang et al. that comprehensively discuss combination therapeutic strategies to overcome PARP-mediated drug resistance in breast and gynecological cancers.

This Research Topic assembles research reports targeting a wide range of cancers and mechanisms centered around overcoming drug resistance by utilizing drug repurposing or therapies using combinations of drugs (Figure 1).
